# A Case of Subacute Thyroiditis following COVID-19 Infection

**DOI:** 10.1155/2022/2211061

**Published:** 2022-05-31

**Authors:** Sameh Samir Elawady, Diwakar Phuyal, Rakesh Kumar Shah, Lubna Mirza

**Affiliations:** ^1^Faculty of Medicine, Tanta University, Tanta, Egypt; ^2^Kirtipur Hospital, PHECT Nepal, Kathmandu, Nepal; ^3^Kathmandu Medical College, Kathmandu, Nepal; ^4^Norman Endocrinology Associates, Norman, OK, USA

## Abstract

*Background*/*Objective*. Since the start of the pandemic, COVID-19 has been associated with several postinfection complications. Subacute thyroiditis (SAT) is an inflammatory disorder of the thyroid that has been reported in the literature following COVID-19 infection. We report a case of SAT following COVID-19 infection. *Case Report*. A 33-year-old female presented with neck pain two weeks after resolution of COVID-19 infection. Her thyroid function tests together with ultrasonographic pictures were consistent with SAT. She was treated with three rounds of medrol dose pack without relief. She then required oral prednisone 40 mg per day and ibuprofen 800 mg once daily for another several weeks that eventually resulted in improvement of her symptoms. *Discussion*. SAT most commonly occurs in females during or after viral infection. The usual course of the disease is hyperthyroidism then hypothyroidism followed by resolution. SAT is clinically diagnosed by lab findings of decreased TSH in the setting of negative thyroid-stimulating and thyroid peroxidase antibodies. All these data are consistent with our case. *Conclusion*. SAT following COVID-19 infection presents with a similar clinical presentation and course as the classic form of SAT, but we should consider the fact that a high-dose corticosteroid treatment might be necessary for such patients.

## 1. Introduction 

Coronavirus disease 2019 (COVID-19) has been rapidly spreading world-wide with over 5 million infected individuals having died through November 26, 2021 [1]. COVID-19 has a clinical presentation varying from asymptomatic to mild upper respiratory tract infection, to severe complications including multiple organ failure especially lungs and kidneys. COVID-19 has been linked to many postinfection complications including subacute thyroiditis (SAT) [2]. Subacute thyroiditis (SAT), also known as de Quatrain thyroiditis, is a self-limiting thyroid disorder linked to some viral infections. It is reported that SAT is linked with several respiratory viruses such as coxsackievirus, mumps, Epstein–Barr virus, cytomegalovirus, and influenza virus [3]. SAT most commonly affects females in their 40s and 50s [4]. It typically presents with fever, myalgia, malaise, pain, and tenderness in the thyroid region, which may radiate to the jaw or neck [5, 6]. The usual course of action is hyperthyroidism, then hypothyroidism followed by resolution within weeks, but some cases may persist for months or may reoccur [7]. The diagnosis of SAT is a clinical diagnosis supported by lab findings of decreased TSH in the setting of negative thyroid-stimulating and thyroid peroxidase antibodies [5]. Here, we present a case of a 33-year-old female patient with post-COVID SAT.

## 2. Case Presentation

**Figure 1:**
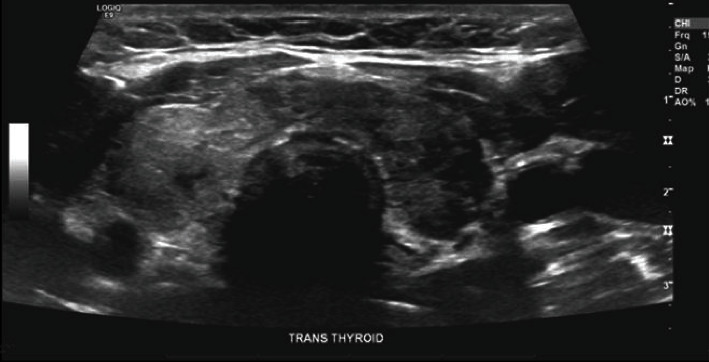
Ultrasound of the thyroid gland showing a heterogeneously enlarged thyroid gland with two small 4 mm solid hypoechoic solid nodules in the isthmus.

A 33-year-old female was referred to endocrinology due to anterior neck pain for four weeks. This pain developed two weeks after contracting COVID-19 infection, which presented with flu-like illness that was confirmed by PCR and resolved within 2 weeks. She initially noticed a swelling and pain in her neck radiating towards the right ear. She also had fever, malaise, and myalgia. She is a nonsmoker, not alcoholic, and is married with four children. Her past medical history included depression, abnormal pap smear, breast biopsies, and pregnancy-induced hypertension. Her medications included duloxetine HCl 30 mg capsule, delayed release particles once a day, and clonazepam 0.5 mg tablet as needed for severe anxiety. On general physical examination, she was alert, oriented, with intact cognitive function. She was cooperative, had good eye contact, judgment, and insight. She had a height of 157.5 cm, weight of 80.74 kg, and body mass index (BMI) of 32.55 kg/m^2^. She had a temperature of 36.8°C, blood pressure of 118/84 mm hg, heart rate of 92 beats/min, and respiratory rate of 12 breaths/min. Head, neck, eye, and extremities examination revealed no abnormalities. Cardiac auscultation showed normal S1, S2, with no murmurs. Lung auscultation was clear. Abdomen was soft, nontender, and nondistended. Neurological examination showed a symmetrical deep tendon reflex of 2+ and good balance. Examination of the neck revealed thyromegaly with tenderness over the anterior neck. Laboratory investigations showed low TSH level (0.04 mU/L), normal free T4, normal free T3, negative thyroid peroxidase antibody (TPO) (0.9 IU/mL), and negative thyroid-stimulating immunoglobulin (TSI) (1.0). Ultrasound of the neck (Figure 1) revealed a heterogeneously enlarged thyroid gland with two small 4 mm solid hypoechoic solid nodules in the isthmus. Right thyroid lobe measured 1.64*∗*4.90*∗*1.69 cm. Left thyroid lobe measured 1.42*∗*4.03*∗*1.38 cm. Isthmus width was 0.49 cm. No abnormal lymph nodes were demonstrated. She was treated with medrol dosepack three times without relief. She continued to feel severe pain with swelling and compressive symptoms requiring oral prednisone 40 mg per day and ibuprofen 800 mg once daily for several weeks. Her symptoms, physical exam, and TSH level normalized within two weeks and steroids were tapered off gradually. An oral consent was obtained from the patient for research and publication, but no written consent has been obtained as there is no patient identifiable data included in this case report.

## 3. Discussion

Thyroiditis refers to heterogeneous groups of disorders that are characterized by some form of thyroid inflammation. SAT is a rare cause of thyroiditis which most commonly occurs two to eight weeks after viral infection. The most common viruses implicated include mumps, measles, influenza, enterovirus, cytomegalovirus, HIV, coxsackievirus, adenovirus, and EBV [3, 5]. SAT could either be due to direct injury by the virus or by the host's immunologic response to the virus [2]. SAT is associated with HLA-B*∗*35, HLA-B*∗*18.01, DRB1*∗*01, and C*∗*04: 01, which confirms genetic predisposition in almost all patients [8].

The prevalence and association of subacute thyroiditis with SARS-CoV-2 varies in the literature. In a prospective study conducted by Bahçecioğlu et al. in Turkey, among 64 patients with SAT, 18.2% of those were associated with SARS-CoV-2. It also concluded that the rate of exposure of SARS-CoV-2 in SAT patients was increased, though the total number of SAT cases pre- and postpandemic period was comparable [9]. In an Italian case series, it was found that, of ten patients referred due to SAT, there were four patients who had evidence SARS-CoV-2 infection [10]. However, another one from Italy concluded that there was no increase in incidence of subacute thyroiditis during the pandemic period in the city of Brescia [11]. A systematic analysis conducted by Giovanella et al., including seven studies and 1237 COVID-19 patients, shows that thyroid dysfunction (defined as abnormal thyroid function tests) varies among the included studies between 13 and 64% [12].

The exact mechanism by which COVID-19 causes SAT is not known, but several explanations have been proposed including induction of an inflammatory response supported by inflammatory infiltration and apoptotic cells inside the thyroid gland [13]. This systemic immune and inflammatory response includes activation of coagulation and complement system, with elevation of many inflammatory cytokines such as IL-6, IL-1*β*, and TNF-*α* [14]. Another possible mechanism is the interaction with ACE2 receptors and transmembrane protease serine 2 (TMPRSS2) receptors, which are abundant in the thyroid tissue and at the same time coronavirus has a high affinity towards [15].

Our patient was diagnosed with SAT based on neck swelling, tenderness, low TSH, negative antibodies, ultrasonographic picture, and disease resolution after treatment with corticosteroids. Our patient have not undergone CRP or ESR testing, which is considered a limitation of our case report as it is considered as one of the main diagnostic criteria of SAT according to Stasiak and Lewiński [16].

A systematic review [2] including 19 articles which included 27 patients with SAT between May 21, 2020, and April 14, 2021, showed that about 83.3% SAT occurred after COVID-19 after a median of 30 days. 12.5% of patients had COVID-19 and SAT concurrently, and in one patient (4.2%), SAT preceded COVID-19 infection. Our patient had SAT after four weeks of COVID-19 and fitted in the group with the majority of the cases. In the study, overt thyrotoxicosis with a decrease in TSH was present in all cases with available reports, which was also the case with our patient who presented with low thyroid-stimulating hormone (TSH) of 0.04 mU/L. Thyroid ultrasound in our patient and most of the patients of the systematic review (20/27) had patchy hypoechogenic areas. Most of the patients in the systematic review had resolution after steroid treatment, with prednisone being the most commonly prescribed steroid at a median dose of 25 mg (IQR 25–35). In contrast to this, SAT in our patient relapsed even with three courses of medrol pack, and required treatment with high-dose prednisone 40 mg per day with ibuprofen 800 mg.

## 4. Conclusion

SAT after COVID-19 presents with a similar clinical presentation and similar course as the classic form of SAT, but we should consider the fact that high-dose corticosteroid treatment might be necessary for such patients.
